# Role of Mitophagy in Coronary Heart Disease: Targeting the Mitochondrial Dysfunction and Inflammatory Regulation

**DOI:** 10.3389/fcvm.2022.819454

**Published:** 2022-02-04

**Authors:** Mingxuan Liu, Ying Wu

**Affiliations:** School of Nursing, Capital Medical University, Beijing, China

**Keywords:** coronary heart disease, arrhythmia, mitochondria, cardiomyocytes, myocardial injury, ischemia/hypoxia, inflammation, oxidative stress

## Abstract

Coronary heart disease (CHD) is one of the main causes of death worldwide. In the past few decades, several in-depth research on the pathological mechanisms and effective treatment methods for CHD have been conducted. At present, the intervention of a variety of therapeutic drugs and treatment technologies have greatly reduced the burden on global public health. However, severe arrhythmia and myocardial fibrosis accompanying CHD in the later stages need to be addressed urgently. Mitochondria are important structural components for energy production and the main sites for aerobic respiration in cells. Mitochondria are involved in arrhythmia, myocardial fibrosis, and acute CHD and play a crucial role in regulating myocardial ischemia/hypoxia. Mitochondrial dysfunction or mitophagy disorders (including receptor-dependent mitophagy and receptor-independent mitophagy) play an important role in the pathogenesis of CHD, especially mitophagy. Mitophagy acts as a “mediator” in the inflammatory damage of cardiomyocytes or vascular endothelial cells and can clear mitochondria or organelles damaged by inflammation under normal conditions. We reviewed experimental advances providing evidence that mitochondrial homeostasis or mitochondrial quality control are important in the pathological mechanism of CHD. Further, we reviewed and summarized relevant regulatory drugs that target mitochondrial function and quality control.

## Introduction

Coronary atherosclerotic heart disease refers to the occurrence of atherosclerosis (AS) in the coronary artery, which narrows or blocks the coronary artery lumen or microvessels (1, 2). The main symptoms of CHD include chest tightness, chest pain, palpitation, and dyspnea (3); however, some patients may show no clinical symptoms. The main risk factors for CHD are diabetes, hyperlipidemia, and hypertension. In addition, people with high life and work pressure, sedentary people, and people who smoke/drink will have a higher risk of disease (3, 4). Due to the rapid pace of life, many people have an unhealthy lifestyle. Some people's irregular eating habits, overeating, eating foods with high calories, high cholesterol, high sugar, and high salt will also increase the risk of AS or vascular stenosis, thereby increasing the risk of coronary heart disease (5, 6).

The pathological mechanism of CHD is mainly damage to the vascular endothelial structure or function caused by pressure stimulation or external factors (7, 8). After vascular endothelial dysfunction, lipids in the blood are deposited in the intima, forming atherosclerotic pathological products that have existed in the blood vessels for a long time (9). The deposited pathological products will gradually form AS, which will lead to stenosis of the coronary artery or microvascular lumen or block blood flow, resulting in serious ischemia/hypoxia injury of the myocardium or microvessels (10, 11). Therefore, AS caused by various factors is one of the main causes of CHD (12, 13), and CHD caused by AS can also be regarded as an inflammatory disease.

Compelling evidence shows that inflammatory injury is inseparable from mitochondrial injury (14, 15). Mitochondria are tiny granular structures with a double-layer membrane found in animal and plant cells and are important for cellular respiration (16–19). In addition to regulating intracellular energy metabolism and cell death, mitochondria can also regulate immune function (20). It has been shown that a vicious cycle of inflammation occurs in mitochondria by changing the levels of metabolites (21, 22), which results in oxidative stress damage to mitochondria leading to mitochondrial structure or function damage, cell death or apoptosis, aging, and various diseases (23, 24). It is also evident from several studies that mitochondrial function activates inflammatory signals directly or indirectly and leads to inflammatory injury of the myocardium or other vascular tissues (25).

In recent years, drugs and basic experimental research targeting mitochondria have become a key hotspot in the field of cardiovascular disease treatment and drug research (26). It has been shown that mitochondrial metabolic dysfunction, mitochondrial quality control imbalance, mitochondrial respiratory chain function defect of cardiomyocytes or coronary vascular endothelial cells, resulting in serious myocarditis injury (26, 27).

This review explains mitophagy and mitochondrial dynamics from the perspective of CHD and myocardial injury (including mitochondrial fission/fusion). It also summarizes the important regulatory mechanism of mitochondrial energy metabolism damage in the pathogenesis and later stages of CHD. In brief, the protective effects of melatonin, coenzyme Q, natural drugs, and Chinese herbal medicine on mitochondrial function through mitophagy and mitochondrial quality control are reviewed. Further, we reviewed and summarized the related therapeutic drugs, which provide a good reference for the research and development of drugs targeting mitochondria.

## Mechanism of Inflammatory Response in CHD

### Inflammation in Acute Myocardial Ischemia/Hypoxia Injury

AS leading to CHD is the concept of an inflammatory process (28). The main pathological basis of coronary AS is the transformation of inflammation from a stable to an unstable state in atherosclerotic plaques (29). Moreover, inflammatory responses have been identified as one of the characteristics of the early stages of CHD, which can progress to the formation of atherosclerotic plaque or myocardial fiber plaque (30). In the past few decades, the cellular effects of different inflammatory factors in the pathogenesis of AS have been confirmed (31), indicating the involvement of inflammatory reactions in the formation of atherosclerotic plaques. Furthermore, the persistence or aggravation of vascular endothelial injury has been reported to activate the immune responses. Mechanistically, the expression of adhesion molecules by vascular endothelial cells mediates the binding and interaction of leukocytes in vascular endothelial cells (32). Leukocytes closely bound to the site of vascular endothelial injury then produce normal stress responses (33). In addition, inflammatory cytokines formed by AS can further guide the migration of adherent leukocytes to the vascular endothelium and promote the proliferation of macrophages in the intimal layer (34). At the same time, the expression of scavenger receptor (SR) is enhanced, resulting in increased macrophage phagocytosis of modified or oxidized lipoprotein particles, the foam cell atheromatous plaques initially formed (34). Furthermore, it has been shown that the shadow of inflammation not only accelerates the progression of AS but also makes the atherosclerotic plaque unstable, making the plaque more likely to rupture, leading to myocardial infarction (MI) or acute myocardial ischemia/hypoxia (35).

Atherosclerotic plaques may also have complications, such as bleeding and intravascular thrombosis, leading to arterial occlusion and acute coronary syndrome (ACS). Moreover, it becomes the pathological basis for narrowing the coronary artery lumen and microvessels in the later stages (36). The unique microenvironment of atherosclerotic plaques is characterized by repeated inflammatory and repair reactions, which are promoted or exacerbated by a variety of inflammatory mediators (37). It has been shown that interleukin (IL)-18/ IL-16, which plays a central role in the inflammatory response, mediates the inflammatory response (38). Further, inflammatory markers not only reflect the stability of atherosclerotic plaques but are also an important index for predicting the risk of CHD (39). For example, the contents of C-reactive protein (CRP), an inflammatory factor, rapidly increase in acute inflammation and other pathological conditions (40). The increased level of CRP mediates the formation of cytokines such as IL-6, produced by granulocytes and macrophages activated by CRP. Its main function is to activate the complement and release inflammatory mediators (41). It can also act on lymphocyte and monocyte receptors, promote the production of lymphokines, enhance the phagocytosis of macrophages, inhibit the aggregation and release of platelets, prevent platelets from causing blood clot contraction, and stimulate the expression of tissue factors on the surface of monocytes and other immune regulatory functions (42). It has been shown that the CRP level is related to the occurrence and severity of inflammation. Reportedly, the serum CRP increases rapidly after injury and decreases after disease recovery (43), suggesting that CRP has a certain predictive effect on clinical practice.

### Inflammation in Myocardial Cell Death

Myocardial cell death or vascular endothelial cell death mediated by an inflammatory response is an important pathological feature of CHD (44). It has been shown that cardiomyocyte death resulting from myocardial injury can cause inflammatory responses (45). In addition, the destruction of organelles, abnormal or damaged protein structure, and metabolites associated with cell death can further activate inflammatory signals, while the infiltration of immune cells and injury-related signals can aggravate the imbalance of intracellular energy metabolism or intracellular oxidative stress, resulting in myocardial injury (46). Accumulating researches have provided evidence that mitochondria are directly and closely associated with cell death and inflammatory responses. Evident also shows that cardiomyocyte death and mitochondrial dysfunction are implicated in various cardiovascular diseases (47).

Additionally, studies have shown that the inflammatory response may act through the mitochondrial pathway (48). Reportedly, inflammation leads to the release of oxidized mtDNA, and oxidized mtDNA binds to the inflammatory bodies (49). mtDNA also activates NOD-like receptor protein 3 (NLRP3). It has been shown that atrial fibrillation associated with CHD is usually related to enhanced inflammatory response mediated by NLRP3 (50). NLRP3 inflammasome inhibitor (MCC950) has also been shown to improve spontaneous atrial premature beats and induce atrial fibrillation in CM-KI mice. Furthermore, in NLRP3 specific knockout mice, sarcoplasmic reticulum Ca^2+^ overload and atrial hypertrophy have been shown to be improved (51).

Studies aimed to develop drugs that inhibit NLRP3 inflammatory corpuscles to improve cardiac inflammatory response have demonstrated that a variety of targeted drugs or traditional Chinese medicine compounds and natural drug active ingredients play a myocardial protective role by regulating NLRP3 inflammatory corpuscles mediated cardiomyocyte apoptosis (52). Inflammatory corpuscles are multi-protein complexes that aggregate and can play an immunomodulatory role in cells and tissues (53). When the inflammation-related enzyme caspase-1 splits, it can provide a defense function. Inflammatory corpuscles containing NLRP3 protein can be formed, and the assembly or start-up procedures of such inflammatory bodies are clear (54). Furthermore, it has also been shown that the binding of lipopolysaccharide (LPS) to TLR4 on the surface of macrophages leads to increased signal transduction of the NF-κB pathway (55), which in turn, causes NLRP3 and IL-1β to increase precursor expression. However, it is unclear when caspase-1 is recruited into inflammatory bodies and how it helps produce inflammatory proteins during the initiation of inflammatory activation reactions (56).

### Inflammation-Induced Oxidative Stress in Acute Myocardial Ischemia/Hypoxia Injury

The interaction between inflammation and oxidative stress plays a crucial role in CHD (57, 58). Oxidative stress occurs throughout the development process of AS from lipid stripe formation to atherosclerotic plaque formation and rupture and mediates vascular endothelial cell dysfunction (25, 59). In addition, inflammation and oxidative stress can induce monocyte spore cells to accumulate in the cell intima and eventually form foam cells (53, 60).

The excessive production of ROS caused by the formation of atherosclerotic plaques is also a feature of endothelial dysfunction (61). In vascular or microvascular endothelial tissue or vascular endothelial cells, LDL can be oxidized (ox-LDL) and then play a role in promoting AS (62). In addition, lipoprotein-related PLA2 (Lp-PLA2) and secretory PLA2 (sPLA2) are generated and modified by inflammatory cells. Lp-PLA2 can hydrolyze the oxidized phospholipids on LDL particles, leading to vascular injury, rupture, or atherosclerotic plaques (63).

Chen et al. found that casein kinase 2-α (CK2-α) mediated oxidative stress injury and mitochondrial homeostasis imbalance are the main causes of cardiomyocyte death and myocardial injury (64). The study demonstrated that overexpression of CK2-α can lead to mitochondrial injury, cardiomyocyte death, and expansion of the myocardial injury area. However, in knockout CK2-α mice (CK2α-CKO), the ischemia-reperfusion (IR) and mitochondrial damages were reduced. In addition, CK2-α can lead to the phosphorylation of fundc1, which can be enhanced by post-transcriptional modification of ser13, which can effectively inhibit mitophagy, resulting in dysfunction of ETC and mitochondrial biosynthesis, severe oxidative stress in cardiomyocytes, and further mediate the abnormal opening of mitochondrial membrane permeability transition pore (mPTP). This process leads to the accumulation of mitochondrial fragmentation and eventually leads to mitochondrial apoptosis.

Moreover, it has also been shown that interferon gene stimulator (STING) can also regulate inflammation/oxidative stress injury and immune response (65). For example, Tang et al. have shown that the survival rate and cardiac function in STING knockout (STING-CKO) mice were significantly improved. Cell experiments further showed that adenovirus overexpression of NLRP3 could counteract the protective effect of STING knockdown on LPS-induced cardiomyocytes. The formation of inflammatory bodies eventually leads to cardiomyocyte injury (66).

### Inflammation-Induced Oxidative Stress in the Progress of Myocardial Cell Death

The NLRP3 inflammatory body is an intracellular multi-protein complex that can regulate the activation of caspase-1 and the effective inflammatory cytokine interleukin(IL)-1β and trigger inflammatory cell death (56). It also induces oxidative stress in cardiomyocytes and vascular endothelial cells (67).

NLRP3 inflammasomes respond to various signals, the activation of NLRP3 inflammasome-related immune pathways leads to a more serious NF-κB-mediated inflammatory response. The activation of NLRP3 inflammatory bodies can help the host defense against invading bacteria and pathogens. However, over-activation of inflammatory bodies can lead to inflammation-related tissue damage.

**Figure 1:**
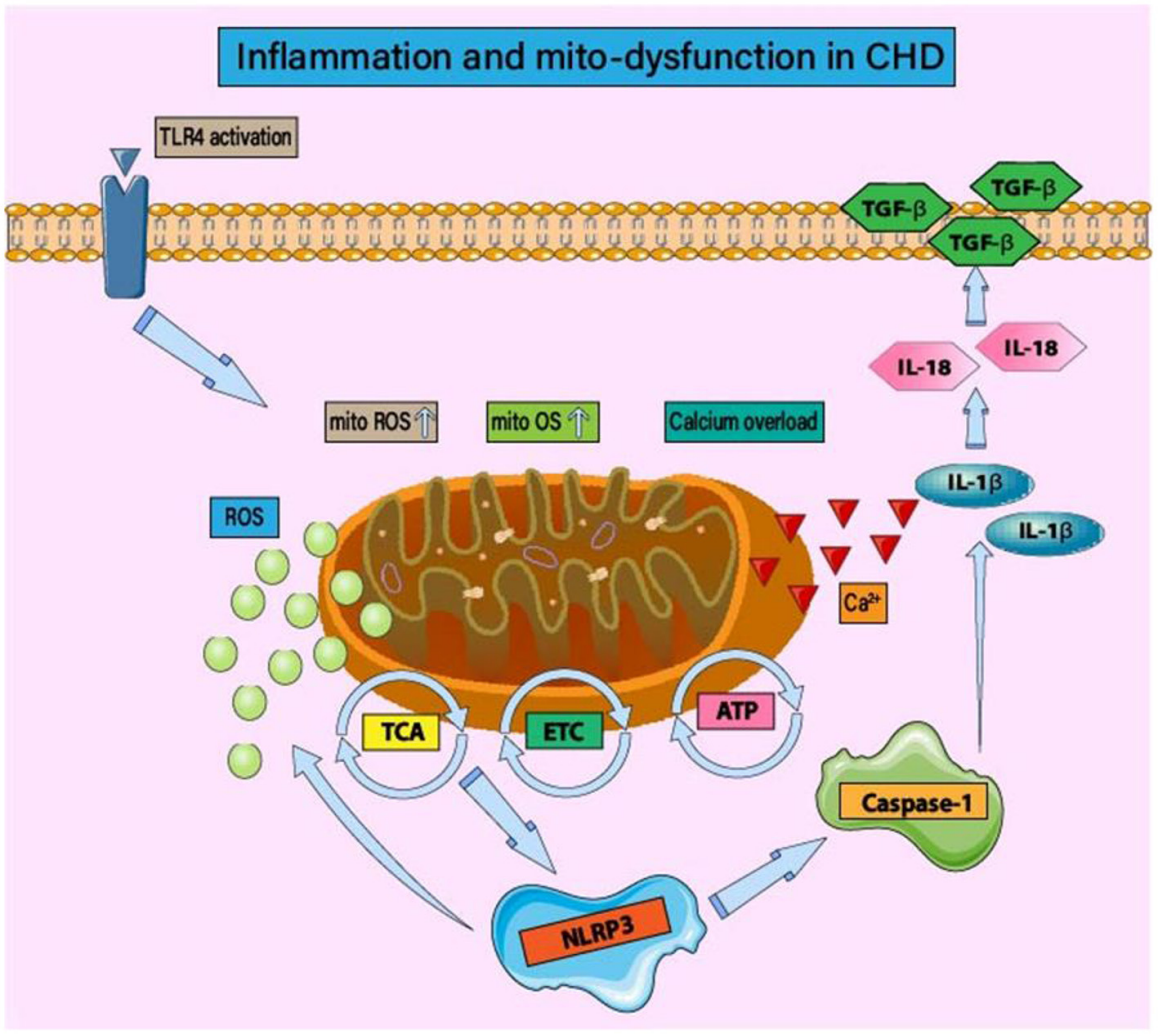
Inflammation and mitochondrial dysfunction in CHD. Under mitochondrial oxidative stress and structural/functional imbalance, the activation of inflammatory bodies is regulated by mitochondrial function. The main mechanism is that the imbalance of mitochondrial homeostasis leads to the production of inflammatory body components NLRP3, caspase-1, and IL-1 β/IL-18. In the early stage of CHD, the activation of TLR4 leads to mitochondrial energy metabolism disorder, tricarboxylic acid cycle disorder, and insufficient ATP production. Mitochondrial dysfunction also affects the regulatory function of ETC. ROS-induced oxidative stress injury is accompanied by calcium homeostasis disorder and calcium overload. The activation of NLRP3 and the occurrence of inflammatory injury lead to the excessive production of ROS and the persistence of the vicious cycle of “inflammation-oxidative stress.” Mitochondrial homeostasis disorder can induce post-translational modification of NLRP3, stabilize NLRP3 in an inactive but activated state of signal transduction, and then affect the activation of inflammatory signal pathways such as NF-κB.

In the late stage of coronary AS leading to vascular stenosis, it is accompanied by severe acute MI, leading to ROS production in ischemic and hypoxic cardiomyocytes (68). As shown in Figure 1, ROS produced by inflammation and acute MI can cause double blows to cardiomyocytes, directly damage the cell membrane, and lead to cell death (64). However, ROS bursts caused by inflammation and oxidative stress stimulate the transcription level and signal transduction of proteins related to mitochondrial quality regulation. It can also act as a host reaction to produce inflammatory cytokines, such as IL-18, IL-1β, and IL-6 in the ischemic myocardial region (69, 70). Furthermore, ROS-induced production of inflammatory cytokines, activation of caspase pathways to stimulate apoptosis, and Ca^2+^ overload can lead to necrosis by enhancing the permeability of the mitochondrial membrane (mitochondrial permeability transition) (71–73).

Tan et al. have demonstrated the protective effect of SERCA on ischemic myocardial microvessels (74). The overexpression of SERCA can reduce coronary microvascular stenosis, inhibit microthrombosis, reduce microvascular inflammatory responses, and improve endothelium-dependent vasodilation. Furthermore, it also improves the viability of coronary microvascular endothelial cells, restores mitophagy, bioenergy, and biogenesis. In contrast, exogenous XO or calcium channel agonists can inhibit or counteract the regulatory effects of SERCA on oxidative stress and calcium release after myocardial ischemia injury.

ROS and inflammatory cytokines can also lead to myofibril sliding, resulting in left ventricular dilation (75, 76). They can also increase collagen deposition and aggravation of myocardial fibrosis and angiogenesis (75, 77). Together, these studies suggested that inflammation and ROS-mediated oxidative stress could be one of the important reasons for ventricular remodeling in the later stages of CHD. Furthermore, Zhou et al. (78) have demonstrated that LPS-induced inflammatory response and oxidative stress can injure HUVECs, leading to a decrease in the expression of SIRT-1 in endothelial cells and the dysfunction of mitochondrial energy metabolism and activation of the mitochondrial apoptotic pathways.

## Mechanism of Inflammatory Response Regulated By Mitophagy in CHD

### Mitophagy in CHD

Mitochondrial quality control mainly maintains the normal activities of mitochondria and cells by regulating the relative stability of mitochondrial quantity. Mitochondrial quality control includes mitophagy, mitochondrial production, and mitochondrial fusion/division. Mitophagy, mediated by different receptors, regulates mitochondrial renewal and degradation by autophagy. Mitochondrial fusion/division is an important factor in mitochondrial repair. Several studies have demonstrated that changes in mitochondrial quantity, morphology, and function affect cardiomyocytes, vascular endothelial cells, and coronary microvascular endothelial cells.

The “autophagy mechanism” of cells won the Nobel Prize in medicine or physiology in 2016. Autophagy refers to the process by which eukaryotic cells phagocytize their cytoplasmic proteins or organelles and encapsulate them into vesicles to form autophagosomes (79, 80). Autophagosomes further fuse with lysosomes to form autophagic lysosomes and degrade their encapsulated contents (81, 82). Maintenance of the balance of energy metabolism and the stability of the cell environment, and regulation of cell renewal, growth, and development are essential physiological processes in a living body. In addition to autophagy, mitophagy is also a physiological process of cell-specific and selective degradation of mitochondria, which plays a significant role in the pathological mechanism of CHD (8, 83). Therefore, the mechanism of mitophagy in CHD myocardial injury has attracted extensive attention since the early 21^st^ century (84–86).

**Figure 2:**
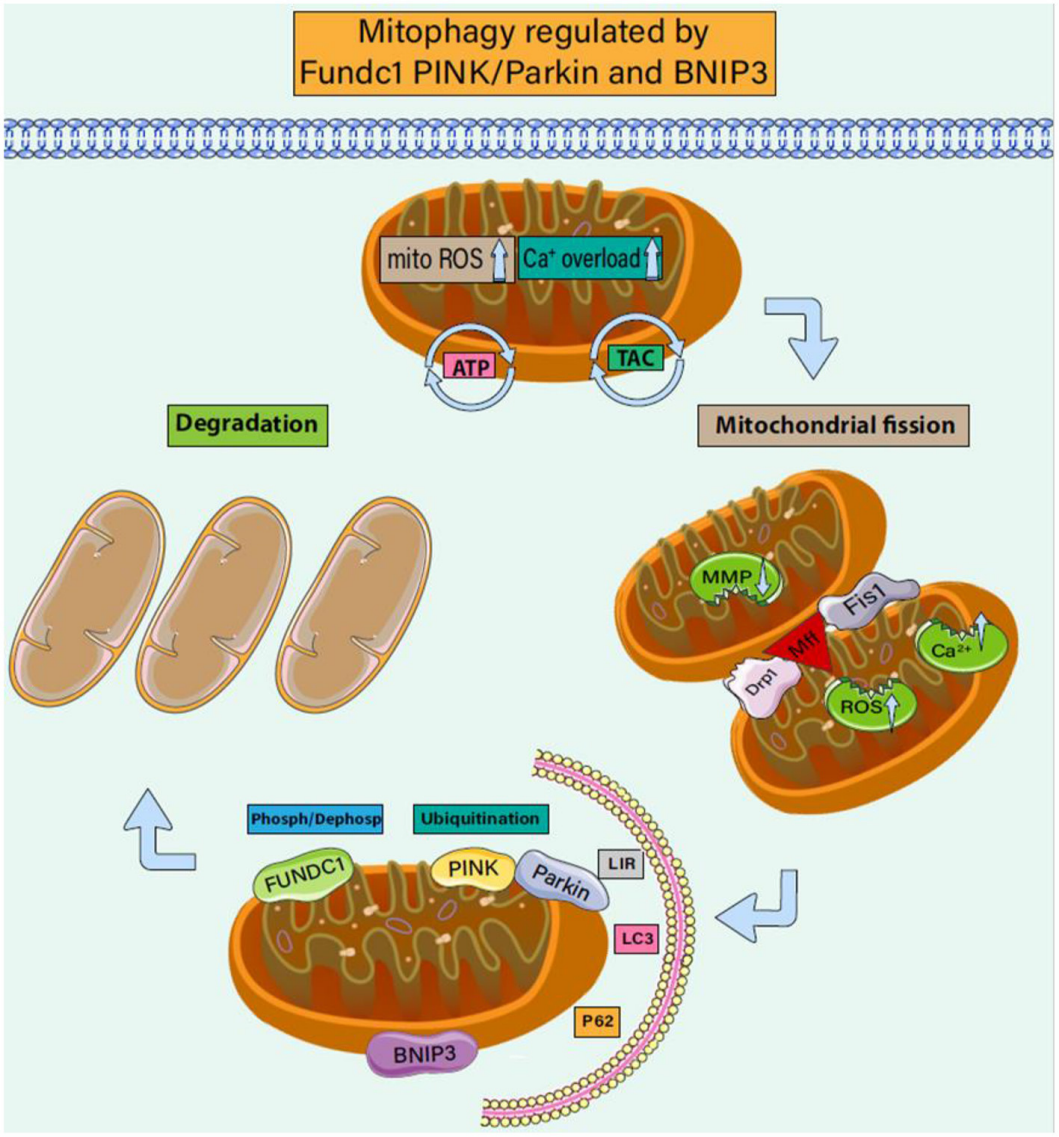
Mitophagy regulated by Fundc1 Pink/Parkin and BNIP3. Mitophagy is a biological process that selectively scavenges damaged mitochondria. It plays an important role in regulating the number of mitochondria in cells, maintaining the structure and function of mitochondria, and maintaining the homeostasis of cell energy metabolism. It is also an important mechanism for the quality control of mitochondria and plays an important role in the process of CHD accompanied by cardiomyocyte injury. Under CHD-induced stress, mitochondrial membrane potential decreases and ROS is overproduced, and the endoplasmic reticulum inputs more calcium ions into mitochondria. Driven by mitochondrial fission proteins drp1, MFF, and FIS1, mitochondria split into two sub mitochondria. Under the phosphorylation (dephosphorylation) of Fundc1 and the receptor regulation of BNIP3, the dysregulated daughter mitochondria react with LC3/LIR to form autophagic lysosomes. The ubiquitination of PINK/Parkin also mediates the formation of autophagic lysosomes. Under the regulation of mitophagy, damaged mitochondria are further cleared.

In addition to their central role in metabolism, mitochondria also play key roles in regulating inflammatory responses (87–89). Mitochondrial fission separates daughter cell mitochondria with damaged membrane potential from healthy mitochondria and experiences a continuous cycle of division and fusion (90). The fission and fusion events lead to an increase or decrease in the mitochondrial membrane potential of the two daughter cell groups (91, 92). The stability of the mitochondrial internal environment requires a perfect balance of mitophagy. The regulation mode of mitophagy can be divided into receptor-regulated mitophagy and non-receptor-regulated mitophagy (93). As shown in Figure 2, these two regulation modes can be completed jointly under the action of Fundc1, PINK/Parkin, and BNIP3.

### FUNDC1-Dependent Mitophagy in CHD

Fundc1 mediated mitophagy has attracted much attention in CHD/acute MI or ischemia/reperfusion injury in recent years (94).

The mitophagy receptor molecule, fundc1, is located on the outer membrane of mitochondria (95, 96). At the N-terminus, it typically interacts with autophagy. Under normal physiological conditions, fundc1 can stably exist in the outer membrane of mitochondria without mediating mitophagy (93). However, under hypoxia, inflammation, or other stress stimuli, fundc1 can recruit autophagy membrane vesicles to wrap mitochondria through LIR and LC3, thus mediating mitophagy (97). Studies have shown that the phosphorylation state of fundc1 regulates mitophagy. Under physiological conditions or in normal myocardium (cardiomyocytes), tyrosine (Tyr18) at position 18 in fundc1 and serine (Ser13) at position 13 near LIR are highly phosphorylated, while Tyr18 and Ser13 show significant dephosphorylation under hypoxic stimulation and FCCP stimulation. Phosphorylation/dephosphorylation of Tyr18 and Ser13 can directly affect the interaction mechanism between FUNDC1 and LC3 and thereby affect mitophagy (98).

Ripk is an upstream signal of Caspase-8 mediated apoptosis. Ripk3-mediated Necroptosis and mitochondria-mediated apoptosis are the main types of cell death in myocardial ischemia. Zhou et al.'s and other related studies on FUNDC1 and myocardial ischemia injury indicate that Ripk3 can affect mitophagy regulated by FUNDC1 phosphorylation or dephosphorylation (99). It has been shown that Ripk3 deficient (Ripk3^−/−^) mice had relatively low apoptotic cells in the infarct area decreased area of MI. Furthermore, inhibition of mitophagy under ripk3 deficiency can enhance cardiomyocyte and endothelial cell apoptosis, increase MI area, and induce microvascular dysfunction. Dephosphorylation of fundc1 in the ischemic state can activate mitophagy, which engulfs mitochondrial fragments and cytochrome-C (Cyto-C), thus blocking the signal of apoptosis. However, reperfusion injury increases the expression of ripk3, which destroys the activation of fundc1, reduces mitophagy and increases the level of apoptosis. Ripk3 promotes mitochondria-mediated apoptosis by inhibiting fundc1 dependent mitophagy in cardiac IR injury.

In a separate study, Zou et al. (100) have demonstrated that the specific binding of fundc1 and P3R2 can regulate the release of Ca^2+^ into the mitochondria and cytoplasm. In addition, the interruption of the interaction between fundc1 and IP3R2 reduces the level of Ca^2+^ in the mitochondria and cytoplasm, resulting in calcium overload, abnormal mitochondrial fission, increased mitochondrial fragmentation, mitochondrial dysfunction, cardiac dysfunction, and myocardial injury. The results showed that FUNDC1 could be located in the MAM by binding with IP3R2 in mouse neonatal cardiomyocytes and intact hearts. Knockout of fundc1 could increase the level of Ca^2+^ in the endoplasmic reticulum, while overexpression of fundc1 decreased the level of Ca^2+^ in the endoplasmic reticulum and disrupted the mitochondrial function. Moreover, the study also showed that FIS1 knockdown eliminated the enhancement of mitophagy mediated by fundc1 overexpression. It is worth noting that knockdown of drp1 cannot reduce fundc1 induced mitophagy, so the interaction mechanism between FIS1 mediated mitochondrial fission and fundc1 overexpression mediated mitophagy deserves further study and clarification. It also provides a good reference for studying the interaction mechanism between mitochondrial fission and mitophagy in myocardial injury.

Other studies (64) have reported the upregulation of CK2-α after acute cardiac IR injury. Increased CK2-α has been shown to contribute to mitochondrial injury, while heart-specific CK2-α knockout (CK2-α-CKO) mice were protected from myocardial ischemic injury. CK2-α also enhances the phosphorylation of fundc1 by post-transcriptional modification of ser13, which effectively inhibits mitophagy. In contrast, CK2α-deletion reversed fundc1 mediated mitophagy and provided a survival advantage for myocardial tissue after IR stress.

### PINK/Parkin-Dependent Mitophagy in CHD

PINK1 and parkin-mediated mitophagy are the most complete and clear mechanisms in mammals (101, 102). The regulation mechanism of PINK1 recruitment and activation of parkind1 has always been the focus of research, and PINK1 phosphorylation of parkin leads to the activation of parkin (103).

A study published in Science showed that mitochondrial outer membrane guanosine triphosphatase mitogen (MFN) 2 could mediate Parkin recruitment to damaged mitochondria (104). Through further research, Yusuke (105) also demonstrated that the synergistic effect of mitochondrial division dynamic protein drp1 could maintain the integrity of mitochondrial structure and function in the mouse heart and brain. Mice lacking cardiac drp1 exhibit fatal cardiac impairments. Moreover, knocking down drp1 and parkin at the same time has been shown to aggravate the defect of cardiac function. When mitochondria are damaged (depolarization, increase in unfolded protein, etc.), PINK1 is blocked from entering the inner mitochondrial membrane and accumulates in the outer mitochondrial membrane. During this, parkin is recruited and activated, but the regulatory mechanism after parkin recruitment and activation (especially when mitochondrial fission or mitochondrial damage occurs) needs further clarification. Song et al. (106) studied parkin gene knockout mice and found that the heart function of parkin gene knockout mice is normal, and parkin has a protective effect on cardiac ischemia. Parkin-mediated mitophagy is the dominant factor in the process of myocardial injury caused by the interruption of mitochondrial fission mediated by the cardiomyocyte-specific power-related protein 1 (drp1). Parkin mRNA and protein were upregulated after directed drp1 gene deletion in adult mouse cardiomyocytes. However, parkin mRNA and protein expression levels were lower in the normal mouse hearts. The overexpression of parkin in cardiomyocytes activates mitophagy without side effects. In addition, cardiomyocyte-specific parkin deletion did not cause myocardial injury or damage to cardiac function, suggesting that the regulatory mechanism of parkin-mediated mitophagy in the normal heart is not affected by parkin expression. Conditional knockout of parkin and drp1 in the hearts of adult mice can inhibit the upregulation of parkin in mitochondria of mitochondrion fission-deficient hearts, increase the survival rate of mice, reduce adverse cardiac remodeling, and reduce cardiomyocyte necrosis. These results suggest that the deletion of parkin can inhibit drp1 induced mitophagy. Parkin deletion did not alter the characteristic mitochondrial enlargement of drp1 deficient cardiomyocytes. Knockout of drp1 in adult mouse cardiomyocytes interrupts mitochondrial fission and does not affect mitochondrial fragmentation. It also significantly upregulates parkin, resulting in excessive and fatal myocardial injury.

### BNIP3-Dependent Mitophagy in CHD

BNIP3 and BNIP3/Nix, multifunctional mitochondrial outer membrane proteins, were initially identified as pro-apoptotic proteins (107). Functional regulation of BNIP3 regulates mitochondrial energy metabolism, mitochondrial dysfunction, mitophagy homeostasis disorder, and mitochondrial structural damage (108). BNIP3 is an important component of the Bcl-2 cell death regulator family. In addition, BNIP3 can directly affect the opening and closing of the mitochondrial membrane permeability transition pore (mPTP), mitochondrial oxidative stress, endoplasmic reticulum, and mitochondrial calcium release/overload (109).

In myocardial tissue, the expression of BNIP3 and Nix can promote cardiomyocyte apoptosis and lead to a decline in cardiac function (110, 111). Hypoxia-induced cardiomyocyte death shows characteristics of apoptosis with DNA breakage and destruction of mitochondrial plasma membrane integrity. It can also lead to cytochrome c release and myocyte death. BNIP3 is a HIF1A target gene, BNIP3 on the outer membrane of mitochondria can interact with LC3 processed on the membrane of phagocytes to promote the isolation of mitochondria during autophagy for degradation (112, 113).

BNIP3 in CHD or acute MI can affect the development of myocardial injury and myocardial fibrosis/hypertrophy by mediating cardiomyocyte inflammatory response (114–116). Different inflammation-related proteins or molecules regulate the expression of BNIP3 at the transcriptional and post-transcriptional levels to promote the development of myocardial injury. Reportedly, mitophagy induced by the BNIP3 signaling pathway can produce a protective effect during myocardial ischemia. The results showed that myocardial ischemia increased the expression of HIF-1 α and activated BNIP3, which subsequently triggered mitochondrial-dependent autophagy. The upregulation of BNIP3 expression has been speculated to promote IR injury in SD rat cardiomyocytes. Furthermore, studies have suggested that the expression of BNIP3 siRNA can reduce autophagy in H9c2 cells under stress (112).

Qin et al. (117) also found that dual-specificity protein phosphatase1 (DUSP), a threonine tyrosine bispecific phosphatase, can regulate BNIP3 mediated mitophagy and affect the regulatory mechanism of cardiomyocyte death. DUSP can regulate MAPK, the energy metabolism of cardiomyocytes, and affect mitochondrial homeostasis. The study also showed that cardiac DUSP1 expression was significantly downregulated after acute myocardial ischemia injury. In cardiomyocytes, a decrease in DUSP1 levels under hypoxic stress can also promote the activation of JNK. The activation of JNK accompanied by the phosphorylation activation of BNIP3 results in the activation of mitophagy. The increase in mitophagy significantly consumes mitochondrial energy, affects the function of mitochondrial energy metabolism, and leads to an increase in the apoptosis level of the mitochondrial pathway. However, the overexpression of DUSP1 can further regulate BNIP3 and mitophagy, affect the level of mitochondrial fission and fragmentation, and protect mitochondrial homeostasis and cardiomyocyte function.

## Targeting Pharmacological of Mitophagy (Mitophagy) for the Management of CHD

CHD has become a “modern era epidemic” worldwide with increasing incidence and mortality rates. However, the high incidence rate of CHD, the low cure rate, and many complications have led to difficulties in developing targeted drugs. Mitophagy dysfunction is an important pathological basis for myocardial ischemia and reperfusion injury in CHD (118). Therefore, the regulatory mechanism of mitophagy targeted by FUNDC1, PINK/Parkin, and BNIP3 has been explored as an important target to improve myocardial IR injury (119). Although many frontier studies have discovered several mitochondria-targeted drugs that can effectively reduce myocardial IR injury, few have successfully passed the clinical transformation (120).

Coronary artery occlusion is the main cause of coronary atherosclerotic heart disease (CAD). Transient coronary artery occlusion and myocardial IR further affect myocardial structure and function leading to serious mitochondrial damage during the process (92). Furthermore, coronary artery occlusion can cause severe MI and lead to myocardial remodeling and heart failure (121). Although the corresponding treatment measures before or during surgery can greatly improve myocardial injury (such as cardiac arrest, ischemic preconditioning, and β-receptor blockers), the protective mechanism of ischemic preconditioning is complex (122). Clinical drug treatment mainly involves statins to delay the formation of AS and anti-angina drugs to improve the blood perfusion of the transient ischemic area. Percutaneous coronary intervention or thrombolytic therapy is mainly used to restore blood flow early as pretreatment cannot be used for sudden acute MI (123). Repeated brief reperfusion after ischemia, called ischemic postconditioning, can also protect the heart. Studies have shown that ischemic postconditioning for percutaneous coronary intervention can reduce the size of MI, and its protective mechanism may be related to promoting the opening/closing of the mPTP (124). At present, many promising drugs have been identified, which are worthy of further clinical verification. Mitochondrial homeostasis and the regulatory mechanisms of mitochondrial fission/fusion and mitochondrial biosynthesis, especially under mitophagy and are essential for cardiac function maintenance (125). Therefore, mitophagy or targeted therapeutic drugs for mitochondrial homeostasis may become an important measure for CHD treatment.

### Pharmacological Intervention in Animals

Many natural drugs, including melatonin, coenzyme Q, and resveratrol, play important regulatory roles and have good therapeutic effects in preventing IR injury and myocardial cell death (126–128). The beneficial effects of melatonin on myocardial ischemia or acute MI injury and its potential mechanism have been explored by several studies (129–132). Melatonin has strong antioxidant and anti-inflammatory properties and plays a promising role in preventing IR injury and myocardial cell death (133). In addition, many reports have explored the mechanism underlying the beneficial effects of melatonin on myocardial IR injury (130, 134, 135).

Zhou et al. (136) have reported that after reperfusion, peroxisome proliferator activated receptors in patients with AMI and the expression of PPARγ was significantly downregulated. The decreased PPARγ expression is closely related to the dephosphorylation of fundc1 and the regulatory mechanism of mitophagy, which further leads to the activity of the mitochondrial electron transport chain complex (ETC) and the increase of mitochondrial respiratory dysfunction and ATP production. However, melatonin can restore PPARγ in platelets, strongly inhibit platelet activation, and block mitophagy. In contrast, knockdown of PPARγ in platelets eliminates the regulatory effects of melatonin, which improves the regulatory ability of mitophagy. This further suggests that melatonin may pass through PPARγ -and FUNDC1 mediated mitophagy, and therefore could have a protective effect against myocardial ischemic injury.

Based on PPARγ and FUNDC1, studies have also discussed that melatonin can maintain the regulation of mitochondrial homeostasis under stress conditions through optic nerve atrophy 1 (OPA1)-related mitochondrial fusion and mitophagy. In the stress state of myocardial IR injury, OPA1-mediated mitochondrial fusion and mitophagy are significantly inhibited, accompanied by the expansion of the MI area and cardiac dysfunction. However, melatonin treatment can maintain myocardial function and cell viability through OPA1-related mitochondrial fusion/mitophagy. Melatonin can increase In addition, it maintains mitochondrial energy metabolism and mitochondrial function while blocking the Caspase-9-mediated mitochondrial apoptosis pathway. In contrast, cardiac-specific knockout of OPA1 can eliminate the effect of melatonin on myocardial and mitochondrial homeostasis. Moreover, the blockade of AMPK can further inhibit the expression of OPA1 and impair the protective effect of melatonin against myocardial injury (137).

It can be seen that melatonin has great potential in up-regulating mitophagy and protecting myocardial injury, and its clinical transformation research is also in progress. In addition to melatonin, many natural drugs also play an unprecedented role in preventing and treating various cardiovascular diseases. Several traditional medicine and modern pharmacology studies have shown the potential protective effects of many traditional Chinese medicine/natural drugs against myocardial injury, anti-inflammatory, anti-oxidation, and anti-vascular injury and regulatory effects on mitophagy and mitochondrial homeostasis (138–141).

Quercetin is an effective active ingredient extracted from a variety of medicinal plants or Chinese herbal medicines (142, 143). It has a good therapeutic effect on cardiovascular diseases. Wang et al. (144) have shown that a certain dose of quercetin can significantly improve myocardial ischemia injury and myocardial fibrosis after acute MI, improve cardiac function and ejection fraction, maintain cardiomyocyte activity, improve mitochondrial energy metabolism in cardiomyocytes, and reduce the incidence of heart failure. Further studies have shown that the therapeutic effect of quercetin may be related to SIRT5 mediated de-succinylation of IDH2 and upregulation of mitophagy (144). However, SIRT5 knockdown eliminated the inhibitory effect of quercetin on the inflammatory response of NLRP3 in cardiomyocytes, and the activity of cardiomyocytes was further reduced. This further suggests that quercetin may protect cardiomyocytes through SITR5 and inhibit inflammation.

Resveratrol is a natural potential anti-aging polyphenol compound that is often used as a nutritional supplement to treat cardiovascular diseases (145). Resveratrol also upregulates selective mitophagy in mice. It has been shown that after resveratrol intervention, the myocardial injury, the number of mtDNA, and mitophagy were significantly increased, and the ROS level was significantly reduced (146). Moreover, studies have shown that activation of autophagy can also improve cardiac activity in mice.

In addition to the above natural drugs, coenzyme Q10 is also expected to become an adjuvant therapeutic drug against myocardial injury (147, 148). Coenzyme Q has anti-aging, antioxidant, and anti-inflammatory effects. Coenzyme-Q is mainly used for patients with CHD, metabolic cardiomyopathy, or other elderly patients. Long-term administration of coenzyme Q10 can reduce the damage of statins to the liver, protect the myocardium, and maintain cardiac function. Furthermore, studies have demonstrated that autophagy/mitophagy impairment and ROS-dependent damage aggravate the development of vascular inflammation in mice. However, Coenzyme Q treatment can inhibit inflammation of vascular tissues and release 8-OHdG, an oxidative stress marker, throughout the body (149).

Mito-Q, a mitochondria-targeted antioxidant, can protect the vascular function and structure of C57 mice (150–152); and can inhibit the excessive production of mitochondrial ROS, increase the expression of superoxide dismutase 2 (SOD2), and restore the relative levels of mitochondrial membrane potential (MMP), oxygen consumption rate (OCR), and ATP. In addition, it indirectly regulate PINK1/Parkin mediated mitophagy and mitochondrial fusion/fission mechanisms (153). Collectively, these studies build a good reference for the study of targeted drugs for vascular injury associated with CHD.

### Pharmacological Intervention in Human Clinical Trials

The above studies explain the protective effects of mitochondria-targeted regulatory drugs (mainly targeting mitophagy) on myocardial ischemia or IR injury in animals. At present, the research results show that many mitochondria-targeted drugs have good therapeutic effects, but they fail to pass the human clinical trials (154), which could be attributed to the unsuitability of animal models, the unknown drug-drug interaction, and other factors. Therefore, the safety and effectiveness of targeted drugs need to be further verified in human clinical trials. In addition, at the cell research level, many cardioprotective drugs may block the endogenous protective pathway, and its deep-seated mechanism needs to be further explained in the process of *in-vivo* research.

Apoptotic death of cardiomyocytes or vascular endothelial cells is an important pathological mechanism of CHD (8, 155). Inflammation caused by hypoxia-ischemia injury and oxidative stress injury will further affect the level of autophagy, resulting in an imbalance of cell energy metabolism, imbalance of mitochondrial homeostasis, or imbalance of cell redox balance (156). Therefore, drug therapy targeting cell life processes is the main direction for future research. *In vitro* studies have also found that melatonin-targeted mitophagy can improve cardiomyocyte injury, although its potential mechanism remains unclear. Melatonin can activate SIRT6 and AMPL/PGC-1 α-Akt signaling, enhance mitochondrial biogenesis and mitophagy, and improve myocardial IR injury. SIRT6 knockdown eliminated the protective effect of melatonin on mitophagy and mitochondrial dysfunction, resulting in decreased mitophagy, decreased mitochondrial biosynthesis, and increased mitochondrial fission and fragmentation. Melatonin targeting SIRT6 may be an important strategy for protecting cardiomyocytes from ischemic injury (157). Other studies have shown that melatonin pretreatment can reduce myocardial injury by maintaining myocardial diastolic function and reducing cardiomyocyte death. Melatonin inhibits IP3R phosphorylation and MCU expression, thereby reducing cytoplasmic and mitochondrial calcium overload. Conversely, MCU activation eliminates melatonin-mediated cardioprotection. These findings suggest that melatonin can protect cardiac function from dysregulation of calcium homeostasis by inhibiting the IP3R-MCU signaling (158).

Quercetin has been shown to be effective against oxidative stress and vascular protection, making it a promising candidate drug for the treatment of cardiovascular diseases. In addition, it plays an important role in cardiomyocyte injury. Ji et al. (142) studied the pharmacological effects of quercetin on human cardiomyocytes. The study using hypoxia/reoxygenation(H/R)-pretreated human cardiomyocytes showed that H/R induced excessive ROS, leading to mitochondrial energy metabolism disorder and endoplasmic reticulum stress, accompanied by a decrease in mitophagy level, finally leading to the apoptosis of cardiomyocytes and a decrease in human cardiomyocyte activity. The treatment of H/R-pretreated human cardiomyocytes with quercetin demonstrated the inhibitory effects of quercetin on the excessive production of ROS and oxidative stress injury and endoplasmic reticulum stress mediated via H/R, improved mitophagy, and the vulnerability of human cardiomyocytes through Sirt1/Tmbim6 (142).

In addition to cardiomyocyte injury, AS and the inflammatory response of vascular endothelial cells are also very important in the pathological mechanism of CHD (159, 160). It has been shown that melatonin can reduce calcium deposition and alkaline phosphatase activity, inhibit caspase 3 expression, increase the expression of Mfn2 and mito-LC3II, maintain mitochondrial function, promote mitochondrial fusion through the OPA1 pathway, and improve the level of mitophagy (161). These experimental results verified the protective effect of melatonin on cardiomyocytes.

Puerarin (PUE), an isoflavonoid isolated from the root of Pueraria, has strong antioxidant properties and has been shown to regulate mitophagy (162, 163). In many experimental studies, PUE showed the potential for anti-AS and its protective effect on endothelial cells. PUE can improve mitochondrial energy metabolism by improving mitophagy or inhibiting LPS-induced inflammatory response in HUVECs, thereby regulating the mechanism of oxidative stress reduction balance. Moreover, the protective effect of PUE on HUVECs is closely related to the SIRT-1 signaling pathway (78).

Studies have also shown the protective effects of resveratrol against endothelial dysfunction induced by high fat. Ox-LDL can induce endothelial cell apoptosis, inhibit proliferation, reduce the activity of mitochondrial respiratory complexes I and III, and the level of intracellular antioxidant enzymes, resulting in excessive production of ROS and mitochondrial dysfunction. On the contrary, resveratrol can upregulate BNIP3 related mitophagy, prevent mitochondrial respiratory complex inactivation, maintain mitochondrial membrane potential, and improve endothelial cell activity. Furthermore, resveratrol can inhibit AMPK. It has been shown that HIF1 eliminates resveratrol-mediated mitochondrial redox balance and endothelial activity protection (164). The above experimental results confirm that resveratrol could be a potential therapeutic drug for AS or CHD with vascular endothelial injury.

In addition to the above mitochondrial-targeted therapeutic and natural drugs, there are many plant-based bioactive components, such as terpenoids, flavonoids, unsaturated fatty acids, vitamins and minerals with effective antioxidants properties. Moreover, traditional Chinese medicine compounds also exert antioxidant effects (165). The targeted regulatory mechanisms of these drugs may be excavated on a large scale in the future, but clinical studies related to the safety and effectiveness of these drugs need to be further verified. Although recent efforts on the clinical applications of coenzyme Q, melatonin, and other natural drugs have been carried out, most of these studies are in progress, or the safety is unknown. Although melatonin, coenzyme Q, and Chinese herbal medicine may show better clinical efficacy in treating cardiovascular diseases, the requirement of long experimental cycles and sample shedding might impose research difficulties and inconsistent results.

## Summary and Conclusion

Mitochondrial quality control (including mitophagy) can regulate the morphology and structure of mitochondria under the stimulation of hypoxia, ischemia, inflammation, and high glucose to respond to the physiological needs of cardiomyocytes and ensure the energy metabolism needs of cardiomyocytes under physiological or stress conditions. Mitophagy is an adaptive response system that regulates mitochondrial quality control. Upon disruption of the mitochondrial fusion/fission balance mechanism, mitophagy can remove mitochondria or organelles damaged by stress injury and maintain energy metabolism in cells. The pathological mechanism of CHD is complex and includes AS in the early stage, myocardial ischemia and hypoxia in the middle stage, myocardial fibrosis, and myocardial hypertrophy in the late stage of CHD. Furthermore, during this process, inflammation, oxidative stress, calcium overload, and other mechanisms can damage cardiomyocytes to varying degrees, leading to programmed apoptosis or death of cardiomyocytes and endothelial cells, as well as inflammatory injury or calcification of coronary vessels/microvessels.

Mitophagy plays a multifaceted role in the regulation of myocardial injury—it plays a protective role and regulates the production of ATP in myocardial injury. Mitophagy promotes the clearance of damaged mitochondria, which is an extremely important phenomenon in the process of myocardial ischemia/hypoxia. Although many studies have suggested that mitophagy is an important protective mechanism for mitochondrial function and cardiomyocyte homeostasis, different receptors can trigger mitophagy to varying degrees, promote cell survival, and remove damaged or structurally abnormal mitochondria. However, some experimental studies have shown that ischemia/hypoxia can activate mitophagy, resulting in a serious impact on ATP production. This reduces the protective effect of the heart, resulting in more extensive myocardial injury. It is noteworthy that the activation or regulation of mitophagy also depends on the degree and duration of cell stress. Different levels of mitophagy and the regulatory mechanism of mitophagy at different stages of myocardial injury may be important issues to be clarified in the future.

Mitochondria is the “bridge” of myocardial cell death caused by inflammation, oxidative stress, and calcium overload. When the self-adaptive regulation mechanism of mitophagy is out of control due to excessive stress, apoptosis, or necroptosis of cardiomyocytes and endothelial cells is activated, resulting in cell death. However, there is still a lack of safe and effective mitochondrial-targeted therapeutic drugs in the clinic. Such drugs (including natural drugs) are promising for enhancing cardiac function and improving myocardial ischemia injury in cooperation with conventional drugs. In the future, more basic experimental studies, especially clinical studies, are needed to further verify the therapeutic effect and the effectiveness of these mitophagy-targeted anti-inflammatory drugs or natural antioxidant drugs.

At present, a variety of natural and adjuvant drugs that can directly or indirectly regulate mitochondrial quality control have been further explored. However, the role of dynamic changes in targeted mitophagy and mitochondrial fusion/fission in cell stress adaptation has not been fully clarified. In the future, the relationship between mitochondria and cell energy metabolism from the perspective of mitochondrial morphology, structure, and reconstruction dynamics related to mitophagy should be explored to more comprehensively and deeply understand the health adaptability of mitophagy and mitochondrial dynamic movement, which will also provide an important theoretical basis for the clear targeting of CHD and aging diseases.

## Author Contributions

ML and YW defined the research theme, collated all related articles, and wrote the manuscript. ML searched for the related articles. All authors contributed to the article and approved the submitted version.

## Conflict of Interest

The authors declare that the research was conducted in the absence of any commercial or financial relationships that could be construed as a potential conflict of interest.

## Publisher's Note

All claims expressed in this article are solely those of the authors and do not necessarily represent those of their affiliated organizations, or those of the publisher, the editors and the reviewers. Any product that may be evaluated in this article, or claim that may be made by its manufacturer, is not guaranteed or endorsed by the publisher.
